# The reporting of prognostic prediction models for obstetric care was poor: a cross-sectional survey of 10-year publications

**DOI:** 10.1186/s12874-023-01832-9

**Published:** 2023-01-12

**Authors:** Chunrong Liu, Yana Qi, Xinghui Liu, Meng Chen, Yiquan Xiong, Shiyao Huang, Kang Zou, Jing Tan, Xin Sun

**Affiliations:** 1 Chinese Evidence-Based Medicine Center, West China Hospital, Sichuan University, 37 Guo Xue Xiang, Chengdu, 610041 Sichuan China; 2 NMPA Key Laboratory for Real World Data Research and Evaluation in Hainan, Chengdu, 610041 Sichuan China; 3 Hainan Healthcare Security Administration Key Laboratory for Real World Data Research, Chengdu, China; 4 Department of Obstetrics and Gynecology, and Key Laboratory of Birth Defects and Related Diseases of Women and Children (Sichuan University), Ministry of Education, West China Second University Hospital, Sichuan University, Chengdu, 610041 Sichuan China; 5 Department of Health Research Methods, Evidence, and Impact, McMaster University, Hamilton, Canada; 6 Biostatistics Unit, St Joseph’s Healthcare—Hamilton, Hamilton, Canada

**Keywords:** Prognostic prediction model, Reporting quality, Obstetric care, TRIPOD

## Abstract

**Background:**

To investigate the reporting of prognostic prediction model studies in obstetric care through a cross-sectional survey design.

**Methods:**

PubMed was searched to identify prognostic prediction model studies in obstetric care published from January 2011 to December 2020. The quality of reporting was assessed by the TRIPOD checklist. The overall adherence by study and the adherence by item were calculated separately, and linear regression analysis was conducted to explore the association between overall adherence and prespecified study characteristics.

**Results:**

A total of 121 studies were included, while no study completely adhered to the TRIPOD. The results showed that the overall adherence was poor (median 46.4%), and no significant improvement was observed after the release of the TRIPOD (43.9 to 46.7%). Studies including both model development and external validation had higher reporting quality versus those including model development only (68.1% vs. 44.8%). Among the 37 items required by the TRIPOD, 10 items were reported adequately with an adherence rate over of 80%, and the remaining 27 items had an adherence rate ranging from 2.5 to 79.3%. In addition, 11 items had a report rate lower than 25.0% and even covered key methodological aspects, including blinding assessment of predictors (2.5%), methods for model-building procedures (4.5%) and predictor handling (13.5%), how to use the model (13.5%), and presentation of model performance (14.4%).

**Conclusions:**

In a 10-year span, prognostic prediction studies in obstetric care continued to be poorly reported and did not improve even after the release of the TRIPOD checklist. Substantial efforts are warranted to improve the reporting of obstetric prognostic prediction models, particularly those that adhere to the TRIPOD checklist are highly desirable.

**Supplementary Information:**

The online version contains supplementary material available at 10.1186/s12874-023-01832-9

## Background

In the era of risk-tailored and personalized medicine, prognostic prediction models, aiming to estimate the individual probability of a specific health outcome occurring in the future by integrating multiple predictors [1, 2], have attracted great interest in many medical areas [3–10]. With proper design and implementation, prognostic models may assist health care providers and patients with related decision-making (e.g., changes in unhealthy lifestyle, adoption of active interventions, adjustment of therapeutic plan, or transfer of high-risk patients to tertiary medical intuitions) [11–13].

For end users, the interpretation and use of a prognostic prediction model highly depends on the reporting. Clear, complete reporting would greatly facilitate end-users to properly judge the scientific rigorousness and generalization of a model. The availability of information regarding epidemiological designs, data sources, statistical methods, model performance and presentation of the final model [14–16] would enable readers to appraise the model and make a transparent judgement about its clinical applicability of the model.

To enhance the reporting transparency of prediction model studies, the Transparent Reporting of a multivariable prediction model for Individual Prognosis Or Diagnosis (TRIPOD) guideline was launched in 2015 with detailed explanation and elaboration [16–19]. The Enhancing the QUAlity and Transparency Of health Research (EQUATOR) network has endorsed adherence to the TROPID checklist [20]. In addition, several journals, such as *The BMJ and PLOS Medicine,* require researchers to include a filled-out checklist of the TRIPOD at the time of submission, while others are encouraged to adhere to the TRIPOD [21]. Since the release of the TRIPOD, some investigations using the TRIPOD checklist have demonstrated that the reporting quality of prediction models among several clinical domains is suboptimal [15, 22–24]..

Obstetric care is a highly active medical domain in the development of prognostic prediction models, with an increasing number of obstetric prognostic models published in recent years [25–27]. Many existing models aim to alert patients to adverse obstetric outcomes earlier and to inform treatment or preventive intervention for high-risk individuals in clinical practice. However, the extent to which these models were well reported was unknown, not to speak of their clinical useability. Therefore, we conducted a cross-sectional survey to investigate the reporting of prognostic models in obstetric care with the aims of identifying the key limitations in reporting and improving the usability of the reported models.

## Methods

A systematic search and review of studies relevant to the derivation and/or validation of multivariable prognostic prediction models in the field of obstetric care was conducted in the present study. We excluded studies aiming to assess the effects of specific variables, develop a diagnostic prediction model, evaluate the impact of prediction models, predict the outcomes in the field of gynaecology or reproduction (e.g., in vitro fertilization, gynaecologic tumor, and menopause), and develop models with a single predictor.

### Literature search and study process

The PubMed database was searched to identify eligible literature published in selected journals from January 1, 2011, to December 31, 2020, given that few prediction models were published before 2011. A consistent search period was conducted with our series of studies [28]. The selected journals included six general medicine journals (*NEJM, Lancet, JAMA, BMJ, Ann of Intern Med, and PLoS Med*) as a sample of leading general medicine journals and the top 15% of journals according to the impact factor of the Science Citation Index for Obstetrics and Gynecology in 2016, which was also consistent with our previous study [28]. MeSH terms and free-text keywords were used for literature searching, and the search language was limited to English (Additional file 1).

Paired investigators (C.L.& Y.Q.) were required to independently screen the title and abstract first and then read the full text to select the eligible literature by using a structured and pilot-tested form. Any disagreements were resolved by discussion or adjudicated by a third reviewer (J.T.).

### Data extraction and quality assessment

Following our previous study [28], if more than one prediction model was reported in a study, we chose the primary model claimed by the authors; otherwise, the first reported model was included. Details on a number of general characteristics were extracted, including (1) first author, (2) country of first author, (3) publication year, (4) publication journal, (5) predicted outcome, (6) involvement of any epidemiologist or statistician, (7) number of study sites, (8) study design, and (9) data source.

Reporting quality was assessed by the TRIPOD checklist with 37 possible items for adherence scoring (Additional file 2). Data extraction and scoring rules were based on the “TRIPOD Adherence Assessment Form” (available at https://www.tripod-statement.org/resources/) developed by the related authors aimed at ensuring uniformity in measuring adherence to TRIPOD for any reviewers [29].

Prediction model studies were categorized into one of the following four types during data extraction according to the TRIPOD Adherence Assessment Form: model development (“D”), external validation of an existing model with/without updating (“V”), incremental value of adding one or more predictor(s) to an existing model (“IV”), and development plus external validation of the same model (“D + V”) [29]. Two authors (C.L. and Y.Q.) independently extracted data and scored the adherence to the TRIPOD checklist of the included studies. Of note, the total number of applicable items for studies varies by the type of prediction model study (“D”, “V”, “IV”, and “D + V”) and model design. In addition, six TRIPOD items (5c, 10d, 10e, 11,14b, and 17) might not be applicable for specific studies (Additional file 2). Any disagreements were discussed with a third reviewer (J.T.) to reach an agreement.

### Analysis

The overall adherence by study was calculated by dividing the sum of the adhered items by the total number of applicable items for the related study, and the adherence by item was calculated by dividing the number of studies adhered to the specific item by the number of studies in which the specific item was applicable. Since the TRIPOD does not state the cut-off point for good or poor reporting, we assumed that the reporting quality per study was good when the overall adherence by study was above 60.0%, while reporting quality per item was good when the adherence by item was over 80.0%. To explore the change in reporting completeness after publication of the TRIPOD, publication year was divided by year 2016—1 year after release of the TRIPOD.

In addition, multivariable linear regression analysis was conducted to explore the association between overall adherence per study and five prespecified study characteristics. These included (1) type of study (“D” as the reference group), (2) publication year (2011–2015 vs. 2016–2020), (3) involvement of any epidemiologist or statistician (no vs. yes), (4) number of study sites (monocenter vs. multicenter), and (5) prospective design (no vs. yes).

Qualitative variables were presented by frequency and percentage, while quantitative data were shown by median and interquartile range (IQR). The Mann–Whitney U test and Kruskal–Wallis test were used to compare the overall adherence per study distributed in different types of studies and other key characteristics. All statistical analyses were performed with SPSS 23.0 (IBM Corp, Armonk, NY) and R version 4.0.3.

## Results

**Fig. 1:**
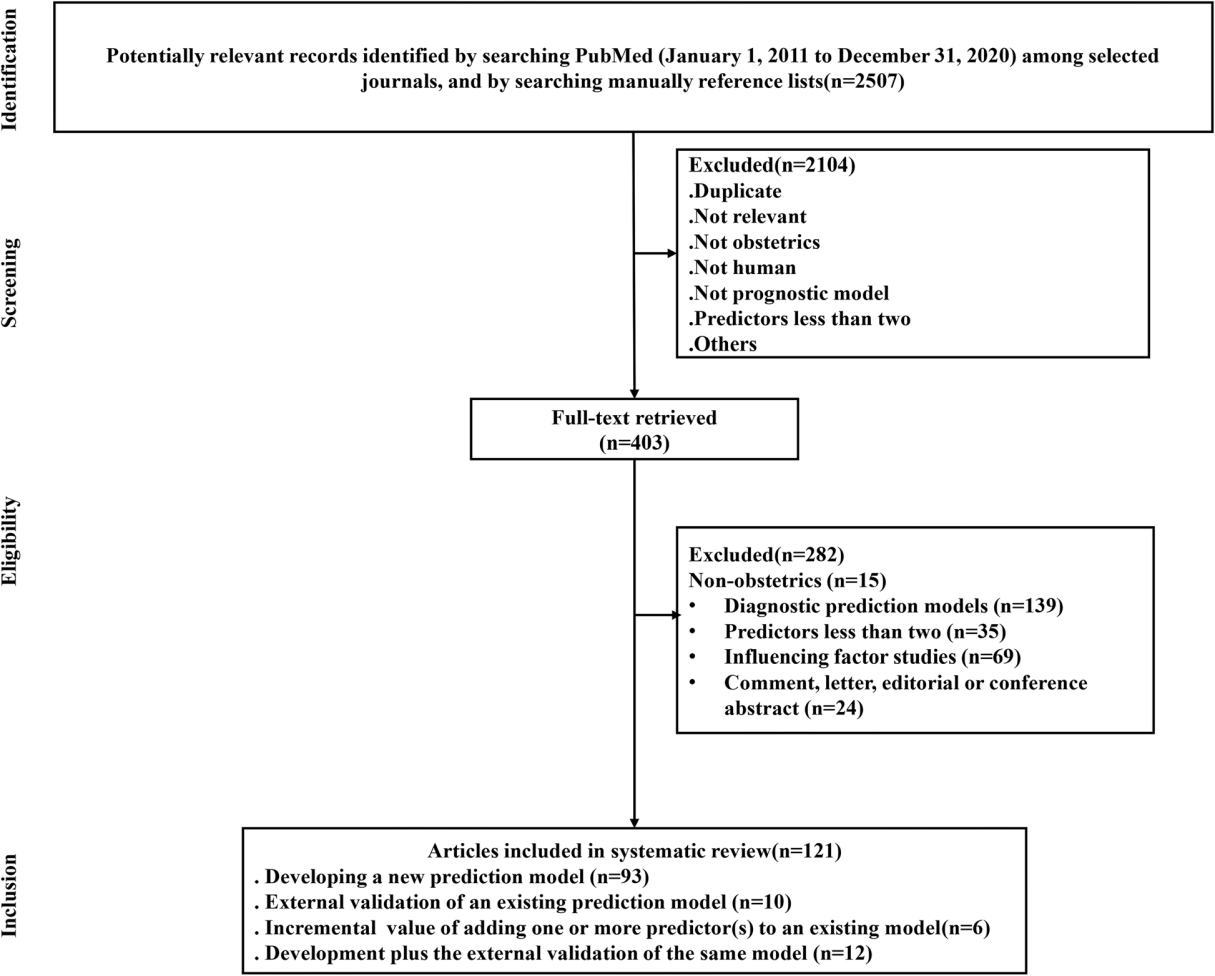
Flow chart of study selection

A total of 2507 records were identified from PubMed, and 121 articles published in 12 journals met the eligibility criteria (Fig. 1). Among the studies, 44 (36.4%) were published before 2016; only 3 (2.5%) were published in the six selected journals; 93 (76.8%) studies developed a new prediction model (“D”), 10 (8.3%) externally validated an existing prediction model (“V”), 6 (5.0%) were about incremental value of adding one or more predictor(s) to an existing model (“IV”), and 12 (9.9%) developed and externally validated the same model (“D + V”); the majority of the publications (97, 80.2%) did not involve any epidemiologist or statistician; more than half of the studies (67, 55.4%) were multicenter studies; 75 (62.0%) were prospective design; 71.9% were cohort studies; preterm delivery (25, 20.7%) and small for gestational age (23, 19.0%) were the two most frequently predicted outcomes (Table 1 and Additional file 3).

**Table 1:** Overall completeness of reporting of obstetrical prediction model studies (median and interquartile range)

Characteristics	N	Adherence (%)^a^	***P value***
**Overall**	121	46.4 (39.3, 54.5)	**–**
**Type of prediction study**			
Model development (type “D”)	93	44.8 (39.3, 51.8)	< 0.001^§^
External validation (type “V”)	10	45.7 (35.6, 65.7)	
Incremental value (type “IV”)	6	49.2 (45.9, 53.3)	
Development and external validation (type “D + V”)	12	68.1 (53.4, 78.2)	
**Publication year**			
2011–2015	44	43.9 (39.3, 55.9)	0.103*
2016–2020	77	46.7 (37.3, 55.9)	
**Involvement of any epidemiologist or statistician**			
No	97	46.4 (39.3, 53.5)	0.630*
Yes	24	45.2 (39.5, 58.7)	
**Number of study sites**			
Monocenter	54	44.4 (39.3, 53.2)	0.277*
Multicenter	67	46.7 (39.3, 55.2)	
**Prospective design**			
No	46	46.4 (40.4, 56.8)	0.665*
Yes	75	46.4 (37.3, 53.1)	

*Note*: § Kruskal–Wallis test;

* Mann–Whitney test

^a^The overall adherence by study was calculated by dividing the sum of the adhered items by the total number of applicable items for related study (%)

### Overall adherence by study

**Fig. 2:**
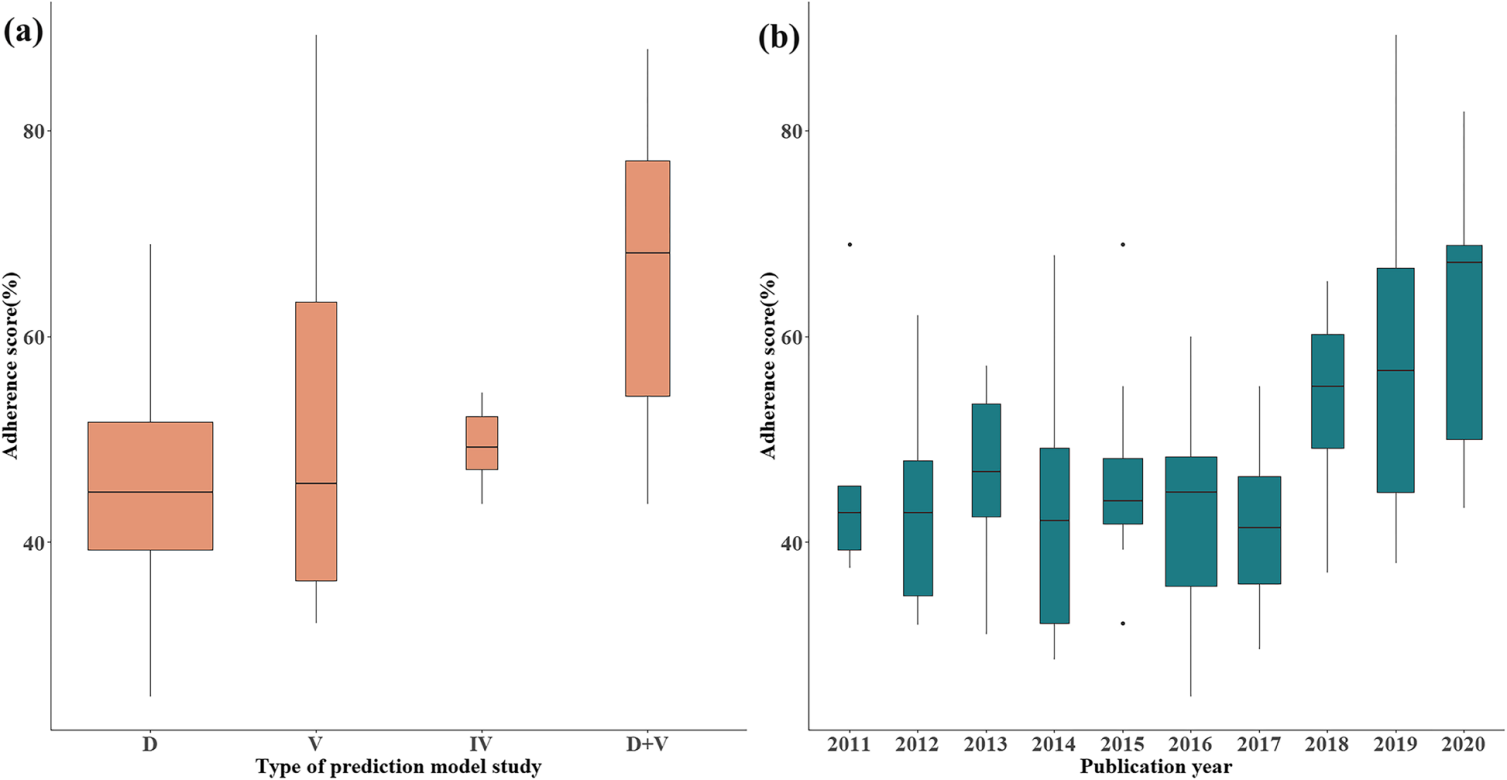
Overall adherence by study among four model types and ten publication years. **a** Four types of predict model study; **b** Publication year

Generally, no study completely adhered to the TRIPOD checklist, and the median overall adherence by study was 46.4%, ranging from 25.0 to 89.3% (IQR [39.3, 54.5%]) (Table 1 and Additional file 3). For different types of prediction model studies, studies of “D + V” showed relatively better adherence to the TRIPOD checklist (68.1%, [53.4, 78.2%]), followed by “IV” (49.2%, [45.9, 53.3%]), “V” (45.7%, [35.6, 65.7%]) and “D” (44.8%, [39.3, 51.8%]), and the differences were statistically significant (*P* < 0.001) (Fig. 2a). The overall adherence by study showed no improvement after 1 year of release of the TRIPOD (2011–2015 vs. 2016–2020, 43.9% vs. 46.7%) (*P* < 0.001), although the adherence from 2018 to 2020 presented a visualized increase depicted in Fig. 2b. In addition, according to univariable analyses, we did not find statistically significant associations between overall adherence by study and study characteristics, including involvement of any epidemiologist or statistician (yes vs. no: 45.2% vs. 46.4%, *P* = 0.630), number of study sites (monocenter vs. multicenter: 44.4% vs. 46.7%, *P* = 0.277), and prospective design (yes vs. no: 46.4% vs. 46.4%, *P* = 0.665) (Table 1).

**Fig. 3:**
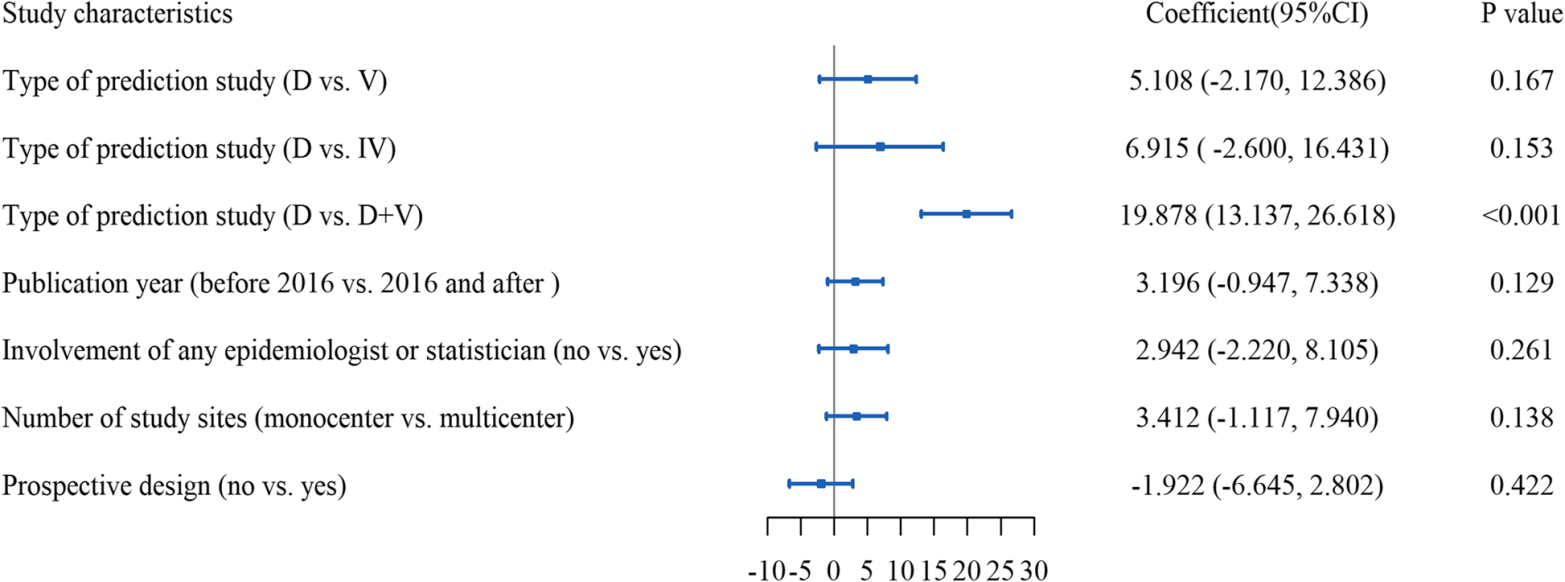
Multivariable linear analysis of potential factors associated with overall adherence measured by the TRIPOD checklist

Furthermore, multivariable analysis also showed that studies of “D + V” were associated with better reporting compared with studies of “D” after adjusting for other factors, but other characteristics showed no statistical association with reporting quality (Fig. 3).

### Adherence by each item

**Fig. 4:**
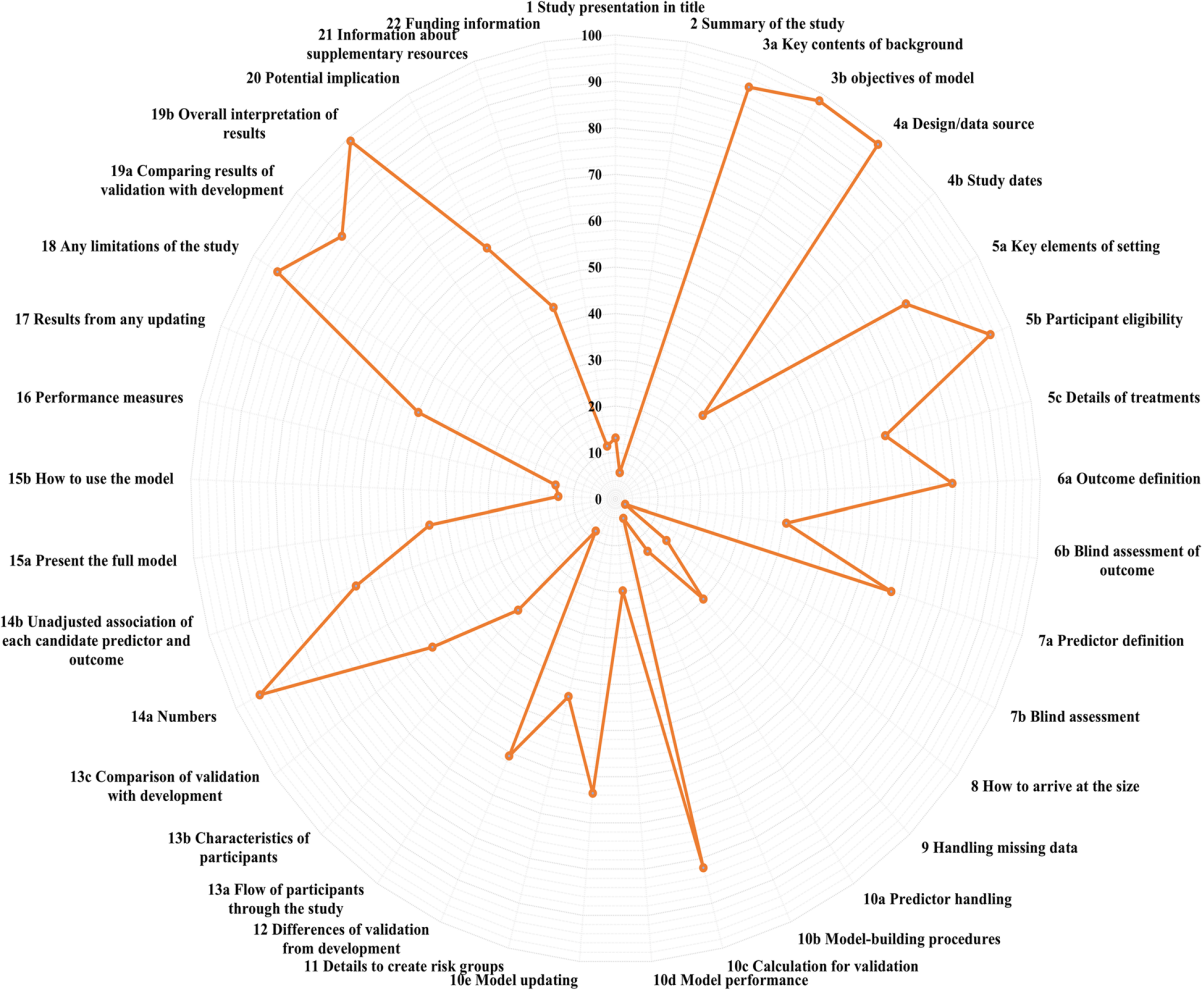
Adherence by item of the TRIPOD checklist in overall. (Note: Because some items were not applicable for all types of prediction studies, the adherence by item was calculated among the number of studies in which the specific TRIPOD item was applicable)

**Fig. 5:**
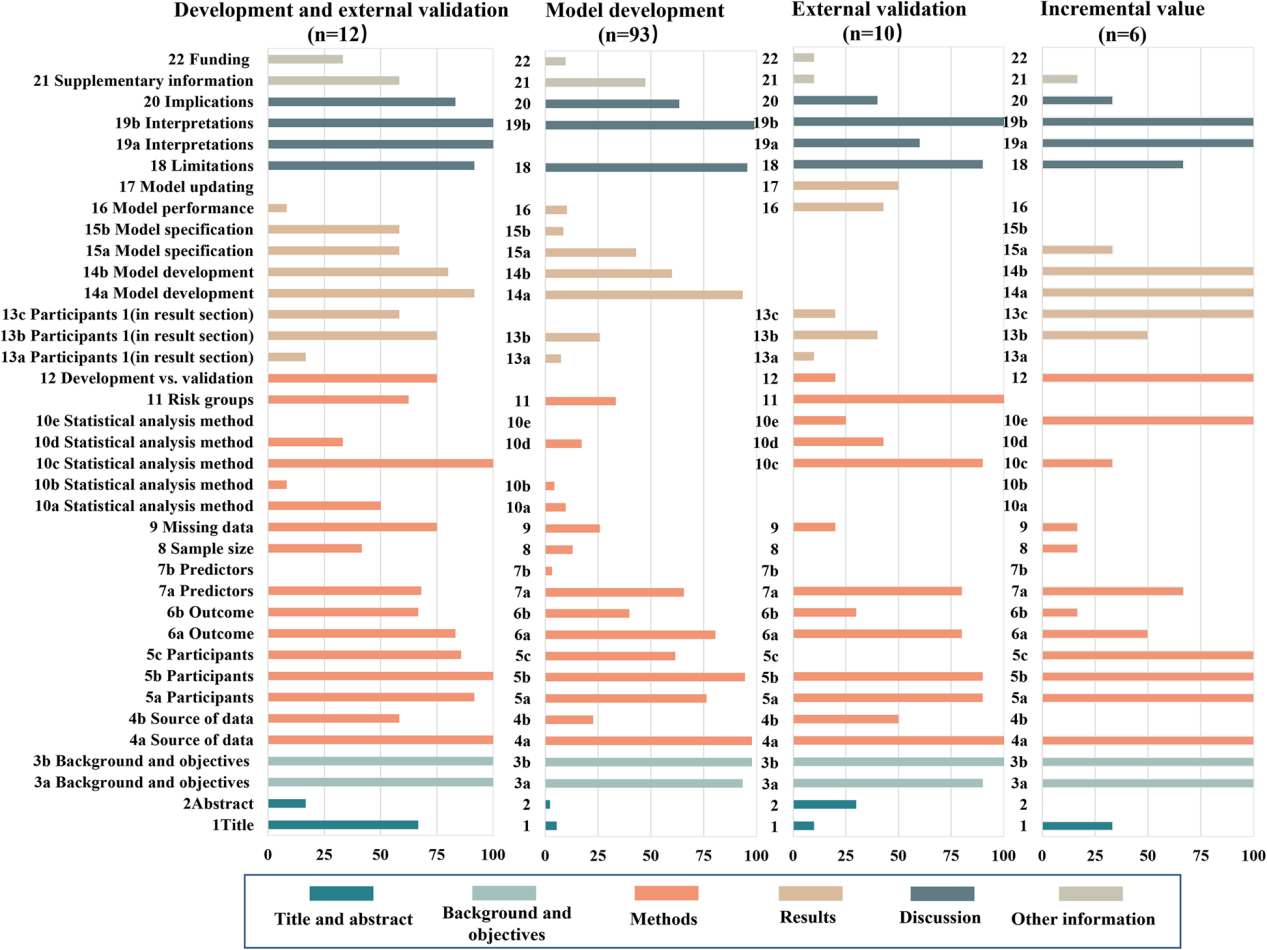
Adherence by item of the TRIPOD checklist among four model type (Note: the items not applicated for a certain model type were not appeared in this figure)

Most studies met part of the requirements of the TRIPOD checklist (Table 2). Among the 37 TRIPOD items, 10 items were reported adequately, with an adherence rate over 80.0%. The proportion of adherence among the remaining 27 items ranged from 2.5 to 79.3%, and 11 items were completely reported in less than 25.0% of the studies. In general, the studies of “D + V” showed the highest completeness. The details are depicted in Figs. 4 and 5.

**Table 2:** Adherence by TRIPOD item among obstetrical prediction model studies, n (%)

Sections	TRIPOD items	Overall	Type of prediction model study			
		(***N*** = 121)	Model development	External validation	Incremental value	Development and external validation
			(***N*** = 93)	(***N*** = 10)	(***N*** = 6)	(***N*** = 12)
**Title**	1 Study presentation in title	16 (13.2)	5 (5.4)	1 (10.0)	2 (33.3)	8 (66.7)
**Abstract**	2 Summary of the study	7 (5.8)	2 (2.2)	3 (30.0)	0 (0.0)	2 (16.7)
**Background and objectives**	3a Key contents of background	114 (94.2)	87 (93.5)	9 (90.0)	6 (100.0)	12 (100.0)
	3b objectives of model	119 (98.3)	91 (97.8)	10 (100.0)	6 (100.0)	12 (100.0)
**Methods**						
Source of data	4a Design/data source	119 (98.3)	91 (97.8)	10 (100.0)	6 (100.0)	12 (100.0)
	4b Study dates	33 (27.3)	21 (22.6)	5 (50.0)	0 (0.0)	7 (58.3)
Participants	5a Key elements of setting	97 (80.2)	71 (76.3)	9 (90.0)	6 (100.0)	11 (91.7)
	5b Participant eligibility	115 (95.0)	88 (94.6)	9 (90.0)	6 (100.0)	4 (100.0)
	5c Details of treatments	24/37 (64.9) ^a^	16/26 (61.5) ^a^	0/2 (0.00)	2/2 (100.0) ^a^	6/7 (85.7) ^a^
Outcome	6a Outcome definition	96 (79.3)	75 (80.6)	8 (80.0)	3 (50.0)	10 (83.3)
	6b Blind assessment of outcome	49 (40.5)	37 (39.8)	3 (30.0)	1 (16.7)	8 (66.7)
Predictors	7a Predictor definition	82 (67.8)	61 (65.6)	8 (80.0)	4 (66.7)	83 (68.0)
	7b Blind assessment	3 (2.5)	3 (3.2)	0 (0.0)	0 (0.0)	0 (0.0))
Sample size	8 How to arrive at the size	18 (14.9)	12 (12.9)	0 (0.0)	1 (16.7)	5 (41.7)
Missing data	9 Handling missing data	36 (29.8)	24 (25.8)	2 (20.0)	1 (16.7)	9 (75.0)
Statistical analysis method	10a Predictor handling	15/111 (13.5) ^a^	9 (9.7)	NA	0 (0.0)	6 (50.0)
	10b Model-building procedures	5/111 (4.5) ^a^	4 (4.3)	NA	0 (0.0)	1 (8.3)
	10c Calculation for validation	23/28 (82.1) ^a^	NA	9 (90.0)	2 (33.3)	12 (100.0)
	10d Model performance	22/111 (19.8) ^a^	15/87 (17.2) ^a^	3/7 (42.9) ^a^	0/5 (0.0) ^a^	4 (33.3)
	10e Model updating	7/11 (63.6) ^a^	NA	1/4 (25.0) ^a^	6 (100.0)	0 (0.0) ^a^
Risk groups	11 Details to create risk groups	18/41 (43.9) ^a^	10/30 (33.3) ^a^	3/3 (100.0)	NA	5/8 (62.5)
Development vs. validation	12 Differences of validation from development	17/28 (60.7) ^a^	NA	2 (20.0)	6 (100.0)	9 (75.0)
**Results**						
Participants	13a Flow of participants through the study	10 (8.3)	7 (7.5)	1 (10.0)	0 (0.0)	2 (16.7)
	13b Characteristics of participants	40 (33.1)	24 (25.8)	4 (40.0)	3 (50.0)	9 (75.0)
	13c Comparison of validation with development	15/28 (53.6) ^a^	NA	2 (20.0)	6(100.0)	7 (58.3)
Model development	14a Numbers	104/111 (93.7) ^a^	87 (93.5)	NA	6 (100.0)	11 (91.7)
	14b Unadjusted association of each candidate predictor and outcome	60/94 (63.8) ^a^	48/80 (60.0) ^a^	NA	4/4 (100.0) ^a^	8/10 (80.0) ^a^
Model specification	15a Present the full model	49/111 (44.1) ^a^	40 (43.0)	NA	2 (33.3)	7 (58.3)
	15b How to use the model	15/111 (13.5) ^a^	8 (8.6)	NA	0 (0.0)	7 (58.3)
Model performance	16 Performance measures	16/111 (14.4) ^a^	9/87 (10.3) ^a^	3/7 (42.9) ^a^	0/5 (0.0) ^a^	4 (33.3)
Model updating	17 Results from any updating	1/2 (50.0) ^a^	NA	1/2 (50.0) ^a^	NA	0 (0.0)
**Discussion**						
Limitations	18 Any limitations of the study	113 (93.4)	89 (95.7)	9 (90.0)	4 (66.7)	11 (91.7)
Interpretations	19a Comparing results of validation with development	24/28 (85.7) ^a^	NA	6 (60.0)	6 (100.0)	12 (100.0)
	19b Overall interpretation of results	120 (99.2)	92 (98.9)	10 (100.0)	6 (100.0)	12 (100.0)
Implications	20 Potential implication	75 (62.0)	59 (63.4)	4 (40.0)	2 (33.3)	10 (83.3)
**Other information**						
Supplementary information	21 Information about supplementary resources (Optional)	53 (43.8)	44 (47.3)	1 (10.0)	1 (16.7)	7 (58.3)
Funding	22 Funding information	14 (11.6)	9 (9.7)	1 (10.0)	0 (0.0)	4 (33.3)

*Note*: *NA* not applicable, there are some TRIPOD items are not applicable for all types of prediction model studies

^a^Percentage is based on number of models for which that item was applicable (and should have been reported). Where this number deviates from the total number of models, the actual number of applicable models is presented as denominator, besides, denominator for the rest of percentage without specification is the total number for overall or each type of prediction models studies

In the title and abstract section, only 16 studies (13.2%) met all requirements of an informative title, with the most poorly reported element indicating the model type (22, 18.2%); only 7 studies (5.8%) completely adhered to all recommendations for abstracts, and the most poorly reported information was model calibration (*n* = 16, 13.2%).

In the methods section, 98.3% of the studies (*n* = 119) well reported study design and source of data, while key study dates, such as start and end of accrual, were poorly reported (*n* = 33, 27.3%). The reporting of definition of outcome and predictors was relatively adequate, 79.3% (*n* = 96) and 67.8% (*n* = 82) of the studies, respectively. However, information on blind assessment of outcome (*n* = 49, 40.5%) and predictors (*n* = 3, 2.5%) was poorly reported. Only 14.9% of the studies (*n* = 18) reported how to arrive at the sample size, while how to handle missing data was described in 29.8% of the studies (*n* = 36). Information regarding statistical analysis methods was generally inadequately reported. Methods to handle predictors (13.5% reported: 15/111) and model-building procedures (4.5% reported: 5/111) had low completeness. Among the 111 studies with categorical outcomes, 19.8% (*n* = 22) reported both discrimination and calibration as measures of model performance owing to low reporting with calibration methods (*n* = 23, 20.7%), while discrimination methods were described in 83.8% of the studies (*n* = 93). Of the 41 studies related to risk groups, 18 (43.9%) described the construction of risk groups in detail. Of 28 studies involving external validation, 17 (60.7%) studies reported the differences in datasets between development and validation (Item 12).

In the results section, the flow of participants through the study was only reported in 10 studies (8.3%), and the characteristics of participants were also poorly described (*n* = 40, 33.1%). Reporting of model specification was insufficient; 44.1% of applicable studies (49/111) clearly presented the final models, while only 13.5% of applicable studies (15/111) explained in detail how to use the models. Among the 111 studies with categorical outcomes, discrimination was reported in 86.5% of the studies (*n* = 96), but calibration was reported in merely 18.0% of the studies (*n* = 20), which resulted in incomplete reporting of whole model performance (*n* = 16, 14.4%). For 2 applicable studies for external validation with updating, only 1 study described the result from updating.

In the discussion and other information section, details of limitation and interpretation were well reported, with adherence rates ranging from 85.7 to 99.2%. Potential implications were reported in 75 studies (62.0%). However, funding information was clearly reported in only 11.6% of the studies (*n* = 14).

## Discussion

### Summary of findings and implications for the future

This cross-sectional survey demonstrated limitations in the reporting of prognostic prediction model studies in obstetrics care based on the TRIPOD checklist. No study adhered to all applicable items, and the median reporting completeness was less than 50%. Furthermore, 11 items were completely reported in less than 25% of studies, especially some essential items for judging models’ clinical acceptance and utilization, such as the modelling-building procedure, the statistical method of predictor handling, presentation of model performance and model specification.

In our survey, less than 15% of studies completely reported information on the title and abstract, which would make it difficult to identify all studies [30]. Key methodological aspects also showed insufficient reporting, such as blind assessment of predictors, rationality of sample size, methods related to model-building procedures, internal validation and performance measures, which made it impossible for readers to understand how model studies were designed and conducted to replicate those studies [31]. Without detailed reporting of information about model specification and performance measures of the final model, end-users, such as clinicians or health-policy-makers, cannot assess the reliability and practical operability of those existing models [32], decreasing the potential clinical application capacity.

In comparison to studies merely developing models, studies with simultaneous development and external validation of the same model had significantly elevated completeness, similar to a survey involving 146 publications in 10 journals with the highest journal impact factor [30]. Since model development, external validation and investigations of impact in clinical practice are the main three phases of prognostic model research [1], we suspected that authors developing and externally validating the same model had more insight into prognostic model research and tended to report details to enhance the chance of clinical use of their models.

The reporting completeness of the studies published between 2016 and 2020 showed no improvement compared with studies published before 2016, consistent with a survey conducted among the top seven general medicine journals [21], but there seems to be an increasing trend of reporting quality in 2018 to 2020. As it may take several years to take effect of a reporting guideline according to experience from other research types, such as CONSORT [33–35], 1 year after the publication of the TRIPOD checklist may be too short to popularize the TRIPOD.

Reporting completeness, indeed, does not reflect the quality of the entire study but has a substantial effect on evaluation and clinical utilization. Insufficient reporting hinders the identification, transformation, and use of all available prognostic prediction models and causes research waste [36, 37]. Reporting guideline-TRIPOD- may be an effective solution. With detailed explanation and elaboration [16], the TRIPOD checklist can act not only as a quality evaluation form but also as guidance for preparing prediction model studies [31]. We strongly suggest that researchers follow the TRIPOD statement not only during the stage of writing up manuscripts about prediction model studies but also during the stage of conception and conducting research, especially for those inexperienced in this study area [38]. In addition, adherence to the TRIPOD checklist could be simultaneously uploaded with manuscripts during the first submission period according to the experiences of reporting guidelines of other research types [39–41]. Among 12 journals in this survey, 3 journals (i.e., *Am J Obstet Gynecol, Obstet Gynecol, PLOS Medicine*) have suggested that prediction model studies adhere to the TRIPOD statement. The median adherence score in 35 studies published in these 3 journals was slightly higher than that in 86 studies among the other 9 journals (51.7% vs. 44.8%, *P* = 0.079). This result suggests an improvement in the reporting quality due to the TRIPOD statement recommendations, although the comparison did not reach statistical significance. Additionally, involvement of a reporting guideline expert in the editorial process may be helpful to improve the completeness of published papers [42]. If there is a word limit in manuscripts in some journals, key information needing detailed elaboration could be reported in the supplementary materials and simultaneously uploaded with main manuscripts.

### Comparison with other studies

Clear and complete reporting of prediction model studies is the foundation for further critical appraisal of the quality and clinical usefulness of models. Unfortunately, incomplete reporting of prediction model studies was found in various medical fields [14, 15, 25, 30, 43]. Among the surveys adopting the TRIPOD checklist as an assessment tool [22, 30, 44, 45], the reporting of obstetric prognostic prediction model studies investigated by our survey was similar to prediction model studies in general medicine (mean adherence of 44.0%) [30], the field of radiomics in oncologic studies (mean adherence of 57.8%) [44], the field of cutaneous melanoma (mean adherence of 61%) [45], and oral health [22]. In addition, deficits in the reporting of prediction models are also found in those surveys. Furthermore, the information of title, abstract, blind assessment of predictors and outcome, sample size, missing data and model development and model performance are the aspects where studies mostly fell short, regardless of field of diseases. Therefore, reporting prediction model studies in nearly all clinical domains may have significant scope for improvement.

### Strengths and limitations

To our knowledge, this is the first study to comprehensively appraise the reporting quality of multivariable prognostic prediction model studies in the field of obstetric care. In this study, the whole process of literature selection, data extraction and synthesis was conducted according to rigid procedures, thus ensuring the representativeness of high-quality prognostic prediction model studies in obstetrics care. In addition, we adopt multivariable analysis to explore the influential factors on reporting completeness.

There were also a few limitations. First, the prediction model studies included in our survey were published in a sample of leading journals about general medicine and obstetrics care; thus, we suspected that the reporting quality of the prognostic prediction model in obstetrics care might be worse than our results. Second, only one model for each study was evaluated, which led to an underreport of the number of existing models in obstetric care. Nevertheless, the objective of this study was to judge the reporting quality of studies. Therefore, the poor reporting status of present models may remain the same.

## Conclusions

In a 10-year span, prognostic prediction studies in obstetric care continued to be poorly reported and had no improvement after the release of the TRIPOD checklist, especially in some essential items evaluating its clinical acceptability and utilization, such as the statistical methods of handling missing data and predictors handling, modelling-building procedure, model performance and model specification. Our findings suggest a strong need to implement TRIPOD checklists in both researchers and journal editors and supplementary materials attached.

## Supplementary Information

**Additional file 1.** Search strategy.

**Additional file 2.** The TRIPOD Checklist.

**Additional file 3.** Basic information and adherence per study.

## Data Availability

The datasets used and/or analyzed during the current study are available from the corresponding author on reasonable request.
