# Supplementary material for: The reporting of prognostic prediction models for obstetric care was poor: a cross-sectional survey of 10-year publications
Source: BMC Med Res Methodol. 2023 Jan 12;23:9. doi: 10.1186/s12874-023-01832-9 (PMC9835271; doi:10.1186/s12874-023-01832-9)
Supplement: Supplementary file 1 — Additional file 1. Search strategy. [file 12874_2023_1832_MOESM1_ESM.docx]

**Additional file 1 PubMed search strategy**

| No. | Search terms |
| --- | --- |
| #1 | pregnan*[tiab] OR obstetric*[tiab] OR woman[tiab] OR caesarean [tiab] OR casarean [tiab] OR caesarian [tiab] OR cesarian [tiab] OR vaginal deliver* [tiab] OR VBAC [tiab] OR placenta praevia[tiab] OR placental abruption [tiab] OR gestational diabetes[tiab] OR GDM[tiab] OR pregnancy induced hypertension[tiab] OR gestational hypertension[tiab] OR eclampsia[tiab] OR preeclampsia[tiab] OR pre-eclampsia [tiab] OR HELLP[tiab] OR post partum haemorrhage [tiab] OR hysterectomy[tiab] OR maternal mortality [tiab] maternal death [tiab] OR anal sphincter rupture[tiab] OR vacuum extraction[tiab] OR forceps extraction [tiab] OR shoulder dystocia[tiab] OR manual placenta removal[tiab] OR cervical incompetence[tiab] OR growth restrict* OR external cephalic version[tiab] OR rupture of membranes[tiab] OR PROM[tiab] OR PPROM [tiab] OR preterm deliver* [tiab] OR preterm labour [tiab] OR preterm labor [tiab] OR preterm birth [tiab] OR fetal distress [tiab] OR shoulder dystocia[tiab] OR Apgar[tiab] OR low birth weight[tiab] OR macrosomia[tiab] OR small for gestational age[tiab] OR large for gestational age[tiab] OR abortion[tiab] OR miscarriage[tiab] OR stillbirth[tiab] OR congenital malformation[tiab] OR birth defect[tiab] OR birth truma[tiab] OR fetal death[tiab] OR neonatal death[tiab] OR perinatal mortality[tiab] |
| #2 | (((((((("risk score"[All fields] OR "prediction model"[All fields] OR "prediction rule"[All fields] OR "risk assessment"[All fields] OR "algorithm"[All fields]))) OR ((Prognostic[tiab] AND (History[tiab] OR Variable*[tiab] OR Criteria[tiab] OR Scor*[tiab] OR Characteristic*[tiab] OR Finding*[tiab] OR Factor*[tiab] OR Model*[tiab])))) OR ((Decision*[tiab] AND (Model*[tiab] OR Clinical*[tiab] OR Logistic Model*[tiab])))) OR (((History[tiab] OR Variable*[tiab] OR Criteria[tiab] OR Scor*[tiab] OR Characteristic*[tiab] OR Finding*[tiab] OR Factor*[tiab]) AND (Predict*[tiab] OR Model*[tiab] OR Decision*[tiab] OR Identif*[tiab] OR Prognos*[tiab])))) OR ((Predict*[tiab] AND (Outcome*[tiab] OR Risk*[tiab] OR Model*[tiab])))) OR ((Validat*[tiab] OR Predict*[ti] OR Rule*[tiab]))) |
| #3 | "0003-4819"[Journal] OR "1549-1676"[Journal] OR "0028-4793"[Journal] OR "0140-6736"[Journal] OR "0098-7484"[Journal] OR "1756-1833"[Journal] OR"1355-4786"[Journal] OR "0029-7844"[Journal] OR "0002-9378"[Journal] OR "0268-1161"[Journal] OR "0015-0282"[Journal] OR "0960-7692"[Journal] OR "0090-8258"[Journal] OR "1470-0328"[Journal] OR "1360-9947"[Journal] OR "2210-7789"[Journal] OR "0378-5122"[Journal] OR "1472-6483"[Journal] |
| #4 | #1 AND #2 AND #3 Filters**: English, from 2011/1/1 - 2020/12/31** |
