# Supplementary material for: The reporting of prognostic prediction models for obstetric care was poor: a cross-sectional survey of 10-year publications
Source: BMC Med Res Methodol. 2023 Jan 12;23:9. doi: 10.1186/s12874-023-01832-9 (PMC9835271; doi:10.1186/s12874-023-01832-9)
Supplement: Supplementary file 3 — Additional file 3. Basic information and adherence per study. [file 12874_2023_1832_MOESM3_ESM.docx]

**Additional file 3 Basic information and adherence to TRIPOD per included study**

| First author | Publication year | Journal | Predicted outcome | Type of predicted outcome | Data source | Model type ^†^ | Total adherence score ^‡^ | Items excluded analysis§ |
| --- | --- | --- | --- | --- | --- | --- | --- | --- |
| Allouche | 2011 | Am J Obstet Gynecol | Preterm Birth | Categorical outcome | Cohort study | D+V | 45.45 | 10e,11,17 |
| Cruz-Martinez | 2011 | Obstet Gynecol | Cesarean section | Categorical outcome | Cohort study | D | 39.29 | 5c,10c,10e,11,12,13c,17,19a |
| Melamed | 2011 | Ultrasound Obstet Gynecol | large for gestational age neonates | Continuous outcome | Cohort study | V | 37.50 | 5c,10a,10b,10d,10e,11,14a,14b,15a,15b,16,17 |
| Peter von Dadelszen | 2011 | Lancet | adverse maternal outcome | Categorical outcome | Cohort study | D | 68.97 | 10c,10e,11,12,13c,17,19a |
| Tsai | 2011 | Obstet Gynecol | Preterm Birth | Categorical outcome | Cohort study | D | 42.86 | 5c,10c,10e,11,12,13c,17,19a |
| Abdelaziz | 2012 | Ultrasound Obstet Gynecol | Pre-eclampsia | Categorical outcome | Cohort study | D | 46.43 | 5c,10c,10e,11,12,13c,17,19a |
| Bastek | 2012 | Obstet Gynecol | Preterm Birth | Categorical outcome | Cohort study | D | 51.72 | 10c,10e,11,12,13c,17,19a |
| Belfort | 2012 | Ultrasound Obstet Gynecol | shoulder dystocia | Categorical outcome | EMR or Chart review | D | 32.14 | 5c,10c,10e,12,13c,14b,17,19a |
| Odibo | 2012 | Ultrasound Obstet Gynecol | fetal growth restriction | Continuous outcome | Cohort study | IV | 46.67 | 5c,10d,11,14b,16,17 |
| Plasencia | 2012 | Ultrasound Obstet Gynecol | Birth weight | Continuous outcome | Cohort study | D | 32.00 | 5c,10c,10d,10e,11,12,13c,14b,16,17,19a |
| Poon | 2012 | BJOG | Preterm Birth | Categorical outcome | Cohort study | D | 35.71 | 5c,10c,10e,11,12,13c,17,19a |
| Schuit | 2012 | BJOG | Cesarean section | Categorical outcome | Secondary analysis of RCT | D | 62.07 | 10c,10e,11,12,13c,17,19a |
| Tsiartas | 2012 | BJOG | Preterm Birth | Categorical outcome | Cohort study | D | 39.29 | 5c,10c,10e,11,12,13c,17,19a |
| Alleman | 2013 | Am J Obstet Gynecol | Preterm Birth | Categorical outcome | Cohort study | D | 42.86 | 5c,10c,10e,11,12,13c,17,19a |
| Bottomley | 2013 | Hum Reprod | Miscarriage or Stillbirth | Categorical outcome | Cohort study | D | 31.03 | 5c,10c,10e,12,13c,17,19a |
| Chaiworapongsa | 2013 | Am J Obstet Gynecol | Pre-eclampsia | Categorical outcome | Cohort study | IV | 54.55 | 5c,11,17 |
| Guha | 2013 | Hum Reprod | Miscarriage or Stillbirth | Categorical outcome | Cohort study | V | 57.14 | 5c,10a,10b,11,14a,14b,15a,15b |
| Huchon | 2013 | Obstet Gynecol | Maternal Mortality | Categorical outcome | Cohort study | D+V | 53.13 | 5c,10e,11,17 |
| Jelliffe-Pawlowski | 2013 | Am J Obstet Gynecol | Preterm Birth | Categorical outcome | Registry dataset | D+V | 43.75 | 5c,10e,11,17 |
| Lindell | 2013 | Ultrasound Obstet Gynecol | large for gestational age neonates | Categorical outcome | Registry dataset | D | 41.38 | 5c,10c,10e,12,13c,17,19a |
| Parra-Saavedra | 2013 | Ultrasound Obstet Gynecol | Cesarean section | Categorical outcome | Cohort study | IV | 50.00 | 11,17 |
| Burkhardt | 2014 | Ultrasound Obstet Gynecol | shoulder dystocia | Categorical outcome | EMR or Chart review | D | 32.14 | 5c,10c,10e,11,12,13c,17,19a |
| Crovetto | 2014 | Ultrasound Obstet Gynecol | small for gestational age neonates | Categorical outcome | Cohort study | D | 41.38 | 10c,10e,11,12,13c,17,19a |
| Cruz-Lemini | 2014 | Am J Obstet Gynecol | infant hypertension and arterial remodeling in intrauterine growth restriction | Categorical outcome | Cohort study | D | 42.86 | 10c,10e,11,12,13c,14b,17,19a |
| de Wilde | 2014 | Hum Reprod | gestational diabetes | Categorical outcome | Cohort study | D | 51.72 | 10c,10e,11,12,13c,17,19a |
| Schwartz | 2014 | Am J Obstet Gynecol | small for gestational age neonates | Categorical outcome | Cohort study | D | 32.14 | 5c,10c,10e,11,12,13c,17,19a |
| Seravalli | 2014 | Am J Obstet Gynecol | small for gestational age neonates | Categorical outcome | Cohort study | D | 28.57 | 5c,10c,10e,11,12,13c,17,19a |
| van Baaren | 2014 | Obstet Gynecol | Preterm Birth | Categorical outcome | Cohort study | D | 48.28 | 10c,10e,12,13c,14b,17,19a |
| Van Oostwaard | 2014 | Pregnancy Hypertens | Pre-eclampsia | Categorical outcome | EMR or Chart review | D | 67.86 | 5c,10c,10e,11,12,13c,17,19a |
| Akolekar | 2015 | Ultrasound Obstet Gynecol | fetal middle cerebral artery pulsatility index (MCA-PI) | Continuous outcome | Cohort study | D | 48.00 | 5c,10c,10d,10e,11,12,13c,14b,16,17,19a |
| Akolekar | 2015 | Ultrasound Obstet Gynecol | Cesarean section | Categorical outcome | Cohort study | D | 32.14 | 5c,10c,10e,11,12,13c,17,19a |
| Bakalis | 2015 | Ultrasound Obstet Gynecol | small for gestational age neonates | Categorical outcome | Cohort study | D | 46.43 | 5c,10c,10e,11,12,13c,17,19a |
| Bredaki | 2015 | Ultrasound Obstet Gynecol | maternal serum alpha-fetoprotein | Continuous outcome | Cohort study | D | 44.00 | 5c,10c,10d,10e,11,12,13c,14b,16,17,19a |
| Buyon | 2015 | Ann Intern Med | adverse perinatal outcome | Categorical outcome | Cohort study | D | 39.29 | 5c,10c,10e,11,12,13c,17,19a |
| Fadigas | 2015 | Ultrasound Obstet Gynecol | small for gestational age neonates | Categorical outcome | Cohort study | D | 39.29 | 5c,10c,10e,11,12,13c,17,19a |
| Figueras | 2015 | Ultrasound Obstet Gynecol | adverse perinatal outcome | Categorical outcome | Cohort study | D | 51.72 | 10c,10e,11,12,13c,17,19a |
| Garcia-Simon | 2015 | Ultrasound Obstet Gynecol | Cesarean section | Categorical outcome | Registry dataset | D | 48.28 | 10c,10e,11,12,13c,17,19a |
| Lesmes | 2015 | Ultrasound Obstet Gynecol | small for gestational age neonates | Categorical outcome | Cohort study | D | 42.86 | 5c,10c,10e,11,12,13c,17,19a |
| Liong | 2015 | BJOG | Preterm Birth | Categorical outcome | Cohort study | D | 40.74 | 5c,10c,10e,11,12,13c,14b,17,19a |
| Mehra | 2015 | Am J Obstet Gynecol | Delivery within 7 days of transvaginal cervical length | Categorical outcome | Cohort study | D | 46.43 | 10c,10e,11,12,13c,14b,17,19a |
| Pay | 2015 | BJOG | Birth weight | Continuous outcome | Registry dataset | D | 44.00 | 5c,10c,10d,10e,11,12,13c,14b,16,17,19a |
| Poon | 2015 | Ultrasound Obstet Gynecol | small for gestational age neonates | Categorical outcome | EMR or Chart review | D | 42.86 | 5c,10c,10e,11,12,13c,17,19a |
| Prick | 2015 | BJOG | need for escape red blood cell transfusion | Categorical outcome | Secondary analysis of RCT | D | 55.17 | 5c,10c,10e,12,13c,17,19a |
| Payne | 2015 | Pregnancy Hypertens | adverse perinatal outcome | Categorical outcome | Cohort study | D | 68.97 | 5c,10c,10e,12,13c,17,19a |
| Akolekar | 2016 | Ultrasound Obstet Gynecol | Miscarriage or Stillbirth | Categorical outcome | Cohort study | D | 39.29 | 5c,10c,10e,11,12,13c,17,19a |
| Akolekar | 2016 | Ultrasound Obstet Gynecol | Miscarriage or Stillbirth | Categorical outcome | Cohort study | D | 46.43 | 5c,10c,10e,11,12,13c,17,19a |
| Aupont | 2016 | Ultrasound Obstet Gynecol | Miscarriage or Stillbirth | Categorical outcome | Cohort study | D | 28.57 | 5c,10c,10e,11,12,13c,17,19a |
| Ayim | 2016 | Ultrasound Obstet Gynecol | ectopic pregnancy | Categorical outcome | Cohort study | D | 46.43 | 5c,10c,10e,11,12,13c,17,19a |
| Bruijn | 2016 | BJOG | Preterm Birth | Categorical outcome | Cohort study | D | 46.67 | 10c,10e,12,13c,17,19a |
| Bruijn | 2016 | Am J Obstet Gynecol | Preterm Birth | Categorical outcome | Cohort study | D | 51.72 | 5c,10c,10e,12,13c,17,19a |
| Crovetto | 2016 | Ultrasound Obstet Gynecol | fetal growth restriction | Categorical outcome | Cohort study | D | 44.83 | 5c,10c,10e,12,13c,17,19a |
| Crovetto | 2016 | Ultrasound Obstet Gynecol | fetal growth restriction | Categorical outcome | Cohort study | D | 44.83 | 5c,10c,10e,12,13c,17,19a |
| Frick | 2016 | Ultrasound Obstet Gynecol | large for gestational age neonates | Categorical outcome | Cohort study | D | 35.71 | 5c,10c,10e,11,12,13c,17,19a |
| Gjessing | 2016 | Ultrasound Obstet Gynecol | Birth weight | Continuous outcome | Registry dataset | D | 40.00 | 5c,10c,10d,10e,11,12,13c,14b,16,17,19a |
| Karlsen | 2016 | Ultrasound Obstet Gynecol | adverse perinatal outcome | Categorical outcome | Cohort study | IV | 48.48 | 5c,11,17 |
| Khalil | 2016 | Ultrasound Obstet Gynecol | Miscarriage or Stillbirth | Categorical outcome | Registry dataset | D | 46.43 | 5c,10c,10e,11,12,13c,17,19a |
| Kuhrt | 2016 | Ultrasound Obstet Gynecol | Preterm Birth | Categorical outcome | Registry dataset | D | 50.00 | 10c,10e,11,12,13c,14b,17,19a |
| Manuck | 2016 | Am J Obstet Gynecol | Preterm Birth | Categorical outcome | Secondary analysis of RCT | D+V | 54.55 | 10e,11,17 |
| Mardy | 2016 | Am J Obstet Gynecol | Vaginal birth after cesarean delivery | Categorical outcome | Cohort study | D | 48.28 | 10c,10e,11,12,13c,17,19a |
| Mastrodima | 2016 | Ultrasound Obstet Gynecol | Miscarriage or Stillbirth | Categorical outcome | Cohort study | D | 35.71 | 5c,10c,10e,11,12,13c,17,19a |
| Miranda | 2016 | Ultrasound Obstet Gynecol | adverse perinatal outcome | Categorical outcome | Cohort study | D | 42.86 | 5c,10c,10e,11,12,13c,17,19a |
| Paternina-Caicedo | 2016 | Am J Obstet Gynecol | Maternal Mortality | Categorical outcome | EMR or Chart review | V | 53.85 | 5c,10a,10b,10e,11,14a,14b,15a,15b,17 |
| Tajik | 2016 | Ultrasound Obstet Gynecol | adverse perinatal outcome | Categorical outcome | Secondary analysis of RCT | D | 60.00 | 10c,10e,12,13c,17,19a |
| Valino | 2016 | Ultrasound Obstet Gynecol | Pre-eclampsia | Categorical outcome | Cohort study | D | 25.00 | 5c,10c,10e,11,12,13c,17,19a |
| Vandermolen | 2016 | Am J Obstet Gynecol | Preterm Birth | Categorical outcome | Cohort study | D | 32.14 | 5c,10c,10e,11,12,13c,17,19a |
| Yerlikaya | 2016 | Ultrasound Obstet Gynecol | Miscarriage or Stillbirth | Categorical outcome | Cohort study | V | 32.14 | 5c,10a,10b,11,14a,14b,15a,15b |
| KUHRT | 2016 | Ultrasound Obstet Gynecol | Preterm Birth | Categorical outcome | Cohort study | D | 48.28 | 5c,10c,10e,12,13c,17,19a |
| Palatnik | 2016 | Am J Obstet Gynecol | shoulder dystocia | Categorical outcome | EMR or Chart review | D | 31.03 | 10c,10e,11,12,13c,17,19a |
| Senapati | 2016 | Fertil Steril | first trimester pregnancy location (ectopic pregnancy vs. miscarriage+viable intrauterine pregnancy) | Categorical outcome | Cohort study | D | 39.29 | 5c,10c,10e,11,12,13c,17,19a |
| Banos | 2017 | Ultrasound Obstet Gynecol | Preterm Birth | Categorical outcome | Cohort study | D | 29.63 | 5c,10c,10e,11,12,13c,14b,17,19a |
| Cavallaro | 2017 | Ultrasound Obstet Gynecol | adverse perinatal outcome | Categorical outcome | Cohort study | IV | 52.94 | 11,17 |
| Eschbach | 2017 | Ultrasound Obstet Gynecol | Severe right ventricular outflow tract obstruction (RVOTO) | Categorical outcome | Cohort study | D | 41.38 | 10c,10e,11,12,13c,17,19a |
| Guy | 2017 | Ultrasound Obstet Gynecol | small for gestational age neonates | Categorical outcome | Cohort study | IV | 43.75 | 5c,11,14b,17 |
| Memtsa | 2017 | Reprod Biomed Online | success of the expectant management | Categorical outcome | Cohort study | D | 34.48 | 10c,10e,11,12,13c,17,19a |
| Miranda | 2017 | Ultrasound Obstet Gynecol | small for gestational age neonates | Categorical outcome | Cohort study | D | 42.86 | 5c,10c,10e,11,12,13c,17,19a |
| Miranda | 2017 | Ultrasound Obstet Gynecol | adverse perinatal outcome | Categorical outcome | Cohort study | D | 46.43 | 5c,10c,10e,11,12,13c,17,19a |
| Pantelis | 2017 | Ultrasound Obstet Gynecol | Preterm Birth | Categorical outcome | Cohort study | D | 32.14 | 5c,10c,10e,11,12,13c,17,19a |
| Parry | 2017 | Am J Obstet Gynecol | small for gestational age neonates | Categorical outcome | Cohort study | D | 37.93 | 10c,10e,11,12,13c,17,19a |
| Perales | 2017 | Ultrasound Obstet Gynecol | Pre-eclampsia | Categorical outcome | Cohort study | D | 46.43 | 10c,10e,11,12,13c,14b,17,19a |
| Poljak | 2017 | Ultrasound Obstet Gynecol | adverse perinatal outcome | Categorical outcome | Cohort study | D | 39.29 | 5c,10c,10e,11,12,13c,17,19a |
| Reboul | 2017 | Ultrasound Obstet Gynecol | small for gestational age neonates | Continuous outcome | Cohort study | V | 36.00 | 10a,10b,10d,10e,11,14a,14b,15a,15b,16,17 |
| Sievert | 2017 | Am J Obstet Gynecol | Cesarean section | Categorical outcome | EMR or Chart review | D | 48.28 | 10c,10e,11,12,13c,17,19a |
| Triunfo | 2017 | Ultrasound Obstet Gynecol | small for gestational age neonates | Categorical outcome | Cohort study | D | 55.17 | 10c,10e,11,12,13c,17,19a |
| Viguiliouk | 2017 | Pregnancy Hypertens | Pre-eclampsia | Categorical outcome | Registry dataset | V | 34.62 | 5c,10a,10b,10e,11,14a,14b,15a,15b,17 |
| Watson | 2017 | Ultrasound Obstet Gynecol | Preterm Birth | Categorical outcome | Cohort study | V | 37.04 | 10a,10b,10e,11,14a,14b,15a,15b,17 |
| Wright | 2017 | Obstet Gynecol | adverse maternal outcome | Categorical outcome | Cohort study | D | 51.72 | 10c,10e,11,12,13c,17,19a |
| Caradeux | 2018 | Ultrasound Obstet Gynecol | small for gestational age neonates | Categorical outcome | Cohort study | D | 39.29 | 5c,10c,10e,11,12,13c,17,19a |
| Griffin | 2018 | Ultrasound Obstet Gynecol | small for gestational age neonates | Categorical outcome | Cohort study | D | 48.28 | 10c,10e,11,12,13c,17,19a |
| Kalafat | 2018 | Am J Obstet Gynecol | Cesarean section | Categorical outcome | Registry dataset | D | 60.71 | 5c,10c,10e,11,12,13c,17,19a |
| Pugh | 2018 | BJOG | Preterm Birth | Continuous outcome | Cohort study | D | 37.04 | 10c,10d,10e,11,12,13c,16,17,19a |
| A. CAVALLARO | 2018 | Ultrasound Obstet Gynecol | adverse perinatal outcome | Categorical outcome | Cohort study | D | 55.17 | 5c,10c,10e,12,13c,17,19a |
| Frederiksen, L. E. | 2018 | Obstet Gynecol | adverse perinatal outcome | Categorical outcome | Registry dataset | D | 55.17 | 5c,10c,10e,12,13c,17,19a |
| Pantelis, A. | 2018 | Ultrasound Obstet Gynecol | Preterm Birth | Categorical outcome | EMR or Chart review | D | 51.72 | 5c,10c,10e,12,13c,17,19a |
| Rahman, S. H. | 2018 | Am J Obstet Gynecol | Postoperative day 1 hematocrit levels after hysterectomy | Continuous outcome | EMR or Chart review | V | 65.38 | 5c,10a,10b,10d,14a,14b,15a,15b,16,17 |
| Sabriá, E. | 2018 | Ultrasound Obstet Gynecol | Pre-eclampsia | Categorical outcome | Cohort study | D | 62.07 | 5c,10c,10e,12,13c,17,19a |
| Sainz, J. A. | 2018 | Am J Obstet Gynecol | Complicated operative delivery | Categorical outcome | Cohort study | D | 58.62 | 5c,10c,10e,12,13c,17,19a |
| Ranjit Akolekar | 2019 | Am J Obstet Gynecol | adverse perinatal outcome | Categorical outcome | Cohort study | D | 37.93 | 5c,10c,10e,12,13c,17,19a |
| John Allotey | 2019 | PLOS Medicine | Seizures | Categorical outcome | Cohort study | D+V | 87.88 | 10e,14b,17 |
| R. O. BAHADO-SINGH | 2019 | Ultrasound Obstet Gynecol | Preterm Birth | Categorical outcome | Registry dataset | D | 41.38 | 5c,10c,10e,12,13c,17,19a |
| A. CIOBANU | 2019 | Ultrasound Obstet Gynecol | small for gestational age neonates | Categorical outcome | Cohort study | D | 44.83 | 5c,10c,10e,12,13c,17,19a |
| Anca Ciobanu | 2019 | Am J Obstet Gynecol | small for gestational age neonates | Categorical outcome | Registry dataset | D | 57.14 | 5c,10c,10e,11,12,13c,17,19a |
| Ciobanu, A. | 2019 | Ultrasound Obstet Gynecol | small for gestational age neonates | Categorical outcome | Cohort study | D | 51.72 | 5c,10c,10e,12,13c,17,19a |
| Harris, B. S. | 2019 | Am J Obstet Gynecol | success of vaginal birth after cesarean delivery | Categorical outcome | EMR or Chart review | V | 66.67 | 5c,10a,10b,10e,14a,14b,15a,15b,17 |
| Ofer Isakov | 2019 | Obstet Gynecol | success in external cephalic version | Categorical outcome | EMR or Chart review | D | 56.67 | 10c,10e,12,13c,17,19a |
| Anshul Jadli | 2019 | Pregnancy Hypertension | Pre-eclampsia | Categorical outcome | Cohort study | D | 55.17 | 5c,10c,10e,12,13c,17,19a |
| Floriane Jochum | 2019 | Obstet Gynecol | Cesarean section | Categorical outcome | Cohort study | D+V | 76.47 | 10e,17 |
| McElrath, T. F. | 2019 | Am J Obstet Gynecol | Preterm Birth | Categorical outcome | Registry dataset | D | 37.93 | 5c,10c,10e,12,13c,17,19a |
| Meertens, L. | 2019 | BJOG | small for gestational age neonates | Categorical outcome | Cohort study | V | 89.29 | 5c,10a,10b,14a,14b,15a,15b,17 |
| Sotiriadis, A. | 2019 | Ultrasound Obstet Gynecol | small for gestational age neonates | Categorical outcome | Registry dataset | D | 58.62 | 5c,10c,10e,12,13c,17,19a |
| J. CARTER | 2020 | Ultrasound Obstet Gynecol | Preterm Birth | Categorical outcome | Registry dataset | D+V | 68.57 | 17 |
| Teresa Cobo | 2020 | Am J Obstet Gynecol | Preterm Birth | Categorical outcome | Cohort study | D+V | 69.70 | 10e,14b,17 |
| Kawakita, T. | 2020 | Obstet Gynecol | Vaginal delivery after preterm induction | Categorical outcome | EMR or Chart review | D+V | 67.65 | 10e,17 |
| Ngwenya, S. | 2020 | Pregnancy Hypertens | adverse maternal outcome | Categorical outcome | Registry dataset | D+V | 78.79 | 5c,10e,17 |
| Nwabuobi, C. | 2020 | Ultrasound Obstet Gynecol | Cesarean section | Categorical outcome | EMR or Chart review | D | 67.86 | 5c,10c,10e,11,12,13c,17,19a |
| Papastefanou, I. | 2020 | Ultrasound Obstet Gynecol | small for gestational age neonates | Categorical outcome | Cohort study | D | 44.83 | 5c,10c,10e,12,13c,17,19a |
| Papastefanou, I. | 2020 | Ultrasound Obstet Gynecol | small for gestational age neonates | Categorical outcome | Cohort study | D | 51.72 | 5c,10c,10e,12,13c,17,19a |
| Papastefanou, I. | 2020 | Ultrasound Obstet Gynecol | small for gestational age neonates | Categorical outcome | Cohort study | D | 44.83 | 5c,10c,10e,12,13c,17,19a |
| Papastefanou, I. | 2020 | Ultrasound Obstet Gynecol | small for gestational age neonates | Categorical outcome | Cohort study | D | 51.72 | 5c,10c,10e,12,13c,17,19a |
| Rizzo, G. | 2020 | Ultrasound Obstet Gynecol | large for gestational age neonates | Categorical outcome | Cohort study | D | 43.33 | 10c,10e,12,13c,17,19a |
| Tsur, A. | 2020 | Ultrasound Obstet Gynecol | shoulder dystocia | Categorical outcome | EMR or Chart review | D+V | 66.67 | 5c,10e,17 |
| Watson, H. A. | 2020 | Ultrasound Obstet Gynecol | Preterm Birth | Categorical outcome | Cohort study | D+V | 81.82 | 5c,10e,17 |

Note: ^†^According to the ***TRIPOD Adherence Assessment Form*** and scoring rules, prediction model studies were categorized into four types: model development ("D"), external validation of an existing model with/without updating ("V"), incremental value of adding one or more predictor(s) to an existing model ("IV"), and the development plus the external validation of the same model ("D+V").

^‡^ Adherence to TRIPOD of each study was calculated by dividing the number of adhered TRIPOD items by the total number of applicable items for that study and expressed as percentage (%)

^§^ TRIPOD item 21 is not taken into account in the overall score in any of the four types of studies, so item 21 was not shown here.
